# Microstructural imaging in temporal lobe epilepsy: Diffusion imaging changes relate to reduced neurite density

**DOI:** 10.1016/j.nicl.2020.102231

**Published:** 2020-02-28

**Authors:** Gavin P Winston, Sjoerd B Vos, Benoit Caldairou, Seok-Jun Hong, Monika Czech, Tobias C Wood, Stephen J Wastling, Gareth J Barker, Boris C Bernhardt, Neda Bernasconi, John S Duncan, Andrea Bernasconi

**Affiliations:** a Department of Clinical and Experimental Epilepsy, UCL Queen Square Institute of Neurology, London,UK; b Epilepsy Society MRI Unit, Chalfont St Peter, UK; c Neuroimaging of Epilepsy Laboratory, McConnell Brain Imaging Centre, Montreal Neurological Institute, McGill University, Montreal, UK; d Department of Medicine, Division of Neurology, Queen's University, Kingston,Canada; e Centre for Medical Image Computing, University College London, London,UK; f NIHR Biomedical Research Centre, UCL Hospitals NHS Foundation Trust/University College London, London,UK; g Department of Neuroimaging, Institute of Psychiatry, Psychology & Neuroscience, King's College London, London,UK; h Lysholm Department of Neuroradiology, National Hospital for Neurology and Neurosurgery, London,UK; i Multimodal Imaging and Connectome Analysis Laboratory, McConnell Brain Imaging Centre, Montreal Neurological Institute, McGill University, Montreal,UK

**Keywords:** Temporal lobe epilepsy, Neurite density, Myelination, Multi-compartment models, Diffusion imaging

## Abstract

**Purpose:**

Previous imaging studies in patients with refractory temporal lobe epilepsy (TLE) have examined the spatial distribution of changes in imaging parameters such as diffusion tensor imaging (DTI) metrics and cortical thickness. Multi-compartment models offer greater specificity with parameters more directly related to known changes in TLE such as altered neuronal density and myelination. We studied the spatial distribution of conventional and novel metrics including neurite density derived from NODDI (Neurite Orientation Dispersion and Density Imaging) and myelin water fraction (MWF) derived from mcDESPOT (Multi-Compartment Driven Equilibrium Single Pulse Observation of T1/T2)] to infer the underlying neurobiology of changes in conventional metrics.

**Methods:**

20 patients with TLE and 20 matched controls underwent magnetic resonance imaging including a volumetric T1-weighted sequence, multi-shell diffusion from which DTI and NODDI metrics were derived and a protocol suitable for mcDESPOT fitting. Models of the grey matter-white matter and grey matter-CSF surfaces were automatically generated from the T1-weighted MRI. Conventional diffusion and novel metrics of neurite density and MWF were sampled from intracortical grey matter and subcortical white matter surfaces and cortical thickness was measured.

**Results:**

In intracortical grey matter, diffusivity was increased in the ipsilateral temporal and frontopolar cortices with more restricted areas of reduced neurite density. Diffusivity increases were largely related to reductions in neurite density, and to a lesser extent CSF partial volume effects, but not MWF. In subcortical white matter, widespread bilateral reductions in fractional anisotropy and increases in radial diffusivity were seen. These were primarily related to reduced neurite density, with an additional relationship to reduced MWF in the temporal pole and anterolateral temporal neocortex. Changes were greater with increasing epilepsy duration. Bilaterally reduced cortical thickness in the mesial temporal lobe and centroparietal cortices was unrelated to neurite density and MWF.

**Conclusions:**

Diffusivity changes in grey and white matter are primarily related to reduced neurite density with an additional relationship to reduced MWF in the temporal pole. Neurite density may represent a more sensitive and specific biomarker of progressive neuronal damage in refractory TLE that deserves further study.

## Highlights

• Widespread changes in diffusion parameters are observed in temporal lobe epilepsy.
• These changes are not specific to the known underlying histological changes.
• Multi-compartment models can quantify neurite density and myelination *in vivo.*
• Grey and white matter diffusion changes correlate with reduced neurite density.

## 1 Introduction

Temporal lobe epilepsy (TLE) is one of the most frequent drug-resistant epilepsies, commonly associated with hippocampal sclerosis, a surgically-amenable lesion (Wiebe et al., 2001). Histopathological studies have also identified widespread neuronal loss and gliosis (Cavanagh and Meyer, 1956; Falconer et al., 1964; Kuzniecky et al., 1987; Nishio et al., 2000) and altered myelination of temporal neocortex (Hardiman et al., 1988; Thom et al., 2000; Kasper et al., 2003; Eriksson et al., 2004). In line with these observations, imaging studies have shown extensive neocortical (Keller and Roberts, 2008; Bernhardt et al., 2010; Bernhardt et al., 2009; Blanc et al., 2011; Labate et al., 2011; Vaughan et al., 2016) and subcortical atrophy (Keller and Roberts, 2008; Bonilha et al., 2010; Bernhardt et al., 2012; Coan et al., 2014; Alvim et al., 2016) indicative of a system-level disorder (Keller et al., 2014; Bernhardt et al., 2015; Vaughan et al., 2016; de Campos et al., 2016). The widespread nature of TLE has been also extensively examined using diffusion weighted MRI, which has shown consistently bilateral alterations of multiple temporal and extra-temporal pathways (Arfanakis et al., 2002; Concha et al., 2005; Gross et al., 2006; Concha et al., 2007; Focke et al., 2008; Yogarajah et al., 2009; Concha et al., 2010; van Eijsden et al., 2011; Otte et al., 2012), as well as the temporo-limbic subcortical white matter (M Liu et al., 2016).

Reduced fractional anisotropy (FA) is thought to result from the combined effects of disruption of myelin sheaths and axonal membranes as well as decreased fibre density (Concha et al., 2012); conversely, increased mean diffusivity (MD) is likely representing widened extra-axonal space associated with reactive gliosis. However, the exact pathophysiological mechanisms remain unclear, as these metrics may be affected by axonal count and density, degree of myelination and fibre organization (Winston, 2012). Further, the tensor model makes the assumption of a single fibre population in each voxel (Jeurissen et al., 2013), even though a given voxel may contain multiple fibre populations with diverse orientations (Jones et al., 2013; Maier–Hein et al., 2017).

Obviating these limitations, recent multi-compartment models represent more than a single tissue type or component in each voxel, thus providing parameters more directly related to neuronal density and myelination. amongst them, NODDI (Neurite Orientation Dispersion and Density Imaging) is an advanced diffusion imaging technique that quantifies neurite (i.e. axons and dendrites) density (Zhang et al., 2012) whereas mcDESPOT (Multi-Compartment Driven Equilibrium Single Pulse Observation of T1 and T2) (Deoni et al., 2008; Deoni et al., 2013) provides estimates of myelin water content through multi-compartment relaxometry.

To improve the understanding of the neurobiological underpinning of TLE, we combined NODDI and mcDESPOT in a surface-based framework (M Liu et al., 2016) and compared the spatial distribution of neocortical grey matter and subcortical white matter anomalies to conventional diffusion-weighted metrics.

## 2 Materials & methods

### 2.1 Participants

We studied 20 patients (mean age 37.1 years, range 23–58 years, 11 men) with medically refractory temporal lobe epilepsy undergoing presurgical evaluation at the National Hospital for Neurology and Neurosurgery, London, United Kingdom. The diagnosis was established by clinical consensus (GPW, AB) from the medical records including history and seizure semiology, 3T structural MRI with an epilepsy protocol, prolonged video EEG telemetry and neuropsychology for all subjects and additional investigations when relevant, including FDG-PET, ictal SPECT or intracranial EEG recordings. Patient demographics and clinical details are summarised in Table 1.

**Table 1:** Patient demographics and clinical characteristics.

Subject	Age/Gender	Age at onset	Duration	MRI	EEG	Other	Pathology
1	33F	9y	24y	R HS	R TL (ii, i)		HS (ILAE type I)
2	30F	4y	26y	R HS	R TL (ii, i)		HS (ILAE type I)
3	58M	51y	7y	R HS	R TL (ii, i)		HS (ILAE type I)
4	34M	7y	27y	R HS	R TL (ii, i)	icEEG – R MTL	HS (ILAE type I)
5	23M	17y	7y	L HS	L TL (ii, i)		HS (ILAE type I)
6	30M	24y	6y	R HS	R TL (ii, i), also L frontopolar (ii)	PET – normal, icEEG – R ant hippocampus	HS (ILAE type I)
7	48F	4y	44y	L HS	L TL (ii, i)		Declined surgery
8	47F	9m	47y	L HS + cerebellar infarct	*L*>*R* TL (ii), no seizures		Declined surgery
9	30M	24y	6y	L HS + precuneus lesion	L TL (ii), L hemisphere (i)		HS (ILAE type I)
10	36F	27y	9y	R HS	R TL (ii,i)		HS (ILAE type I)
11	57M	44y	13y	R HS	Nil (ii), R TL (i)		Declined surgery
12	50M	24y	26y	L HS	L TL (ii, i)		Declined surgery
13	31M	22y	9y	R HS	*R*>*L* TL (ii), R TL (i)	icEEG – R hippocampus	Declined surgery
14	33M	26y	7y	R HS	R TL (ii, i)		HS (ILAE type I)
15	31F	6y	25y	Normal	R TL (i)	PET – R TL	Declined surgery
16	38M	13y	25y	Normal	*R*>*L* TL (ii, i)	PET – R TL SPECT – R TL	Declined icEEG
17	26F	4y	22y	Normal	*R*>*L* TL (ii)	PET – R TL	Undergoing investigation
18	48F	41y	7y	Normal	R TL (ii, i)	PET – normal	Unsuitable for icEEG
19	35F	19y	16y	Normal	B TL (ii), L TL (i)	PET – L TL	Declined icEEG
20	24M	7y	17y	Normal	L TL (ii, i)	PET – L TL	Unsuitable for icEEG

The majority of patients (*n* = 14) had radiological evidence of hippocampal sclerosis (HS) with volume loss and/or signal hyperintensity on T2 or T2-FLAIR supported by quantitative evaluation of hippocampal volumes (Winston et al., 2013) and hippocampal T2 relaxometry (Winston et al., 2017) (Table 2). A comparison of the remaining patients to healthy controls revealed normal hippocampal volumes but prolonged hippocampal T2 relaxation times, more marked ipsilaterally (Student's *t*-test, *p* < 0.0001). Nine patients (45%) have undergone anterior temporal lobe resection (ATLR) and all had evidence of neuronal loss and gliosis predominantly in CA1 and CA4 subfields (ILAE type I) (Blumcke et al., 2013). Two patients with normal neuroimaging were deemed unsuitable for intracranial EEG due to psychiatric comorbidity, one is still undergoing investigation and the remainder declined to proceed.

**Table 2:** Hippocampal volumes and T2 relaxometry.

	Hippocampal volumes (cm^3^)		Hippocampal T2 values (ms)	
	Ipsilateral (controls - L)	Contralateral (controls - R)	Ipsilateral (controls - L)	Contralateral (controls - R)
Controls (*n* = 20)	2.868 (0.193)	2.930 (0.214)	112.6 (3.3)	113.4 (3.6)
Patients (*n* = 20)	2.305 (0.567) [*p* = 0.0002]	2.850 (0.257) [*p* = 0.457]	124.1 (8.0) [*p* < 0.0001]	115.6 (3.3) [*p* < 0.0001]
Patients with HS (*n* = 14)	1.998 (0.316) [*p* < 0.0001]	2.808 (0.2540 [*p* = 0.237]	126.7 (8.1) [*p* < 0.0001]	115.8 (3.7) [*p* < 0.0001]
Patients without HS (*n* = 6)	3.021 (0.286) [*p* = 0.354]	2.948 (0.259) [*p* = 0.676]	118.0 (2.7) [*p* < 0.0001]	115.1 (2.5) [*p* < 0.0001]

Hippocampal volumes (corrected for intracranial volume) and hippocampal T2 relaxation times are given for each group as mean (sd). In the patient subgroups, the p values from a Student's *t*-test comparing against each group against all control hippocampi is given.

A group of 20 age- and sex-matched healthy controls (mean age 37.2 years, range 23–60 years, 11 men) without any history of neurological or psychiatric disease underwent the same neuroimaging protocol. The study was approved by the National Hospital for Neurology and Neurosurgery and the UCL Queen Square Institute of Neurology Joint Ethics Committee, and written informed consent was obtained from all subjects.

### 2.2 Imaging acquisition

MRI studies were performed on a 3T GE MR750 scanner (General Electric, Waukesha, WI, U.S.A.). Standard imaging gradients with a maximum strength of 50 mT/m and maximum slew rate 200 T/m/s were used. All data were acquired using a body coil for transmission and 32-channel phased array coil for reception.

Standard clinical sequences were performed including a 1 mm isotropic volumetric three‐dimensional (3D) T_1_‐weighted inversion‐recovery fast spoiled gradient recalled echo (echo/repetition/inversion time, TE/TR/TI 3.1/7.4/400 ms, field of view (FOV) 224 × 256 × 256 mm, matrix 224 × 256 × 256, parallel imaging acceleration factor 2).

Multi-shell diffusion MRI data were acquired with a 2 mm isotropic single-shot spin echo sequence with a FOV of 256 × 256 mm, matrix 128 × 128 and 70 slices (TR/TE = 7600/74.1 ms; ∂/Δ = 21.5/35.9 ms; parallel imaging acceleration factor 2). A total of 115 volumes were acquired with 11, 8, 32, and 64 gradient directions at b-values of 0, 300, 700, and 2500s/mm^2^ respectively as well as a single *b* = 0-image with reverse phase-encoding.

The acquisition protocol for the mcDESPOT fitting consisted of three sagittally-orientated 3D scans: a spoiled gradient-recalled echo (SPGR), an inversion-recovery SPGR (IR-SPGR), and a balanced steady-state free precession (bSSFP) scan. The FOV was 220 × 220 × 163 mm (APxISxRL) with an acquisition matrix of 128×128×96 corresponding to a voxel size of 1.72 × 1.72 × 1.7 mm. SPGR data was acquired with 8 flip angles (3, 4, 5, 6, 7, 9, 13, 18°), TR/TE 8.3/2.5 ms. The IR-SPGR data were matched to the SPGR data in all parameters but acquired with an inversion time of 450 ms and a single flip angle of 5°. The bSSFP data was acquired with 8 flip angles (12, 16, 21, 27, 33, 40, 51, 68°) each with two phase cycling angles (0 and 180°), TR/TE 4.4/2.2 ms.

### 2.3 Imaging analysis

#### 2.3.1 MRI preprocessing

T1-weighted images were automatically corrected for intensity non-uniformity (Sled et al., 1998) followed by intensity standardization and linear registration to a hemisphere-symmetric MNI ICBM152 template (Fonov VSE et al., 2009) and classification into white matter, grey matter and cerebrospinal fluid (CSF) (Kim et al., 2015).

Diffusion data were corrected for scanner drift (Vos et al., 2017) and eddy current-induced distortions, subject movement and susceptibility-induced distortions using FSL v5.10 eddy and topup (Andersson and Sotiropoulos, 2016; Andersson et al., 2003).

Conventional diffusion tensor imaging metrics (fractional anisotropy [FA], mean diffusivity [MD], axial diffusivity [AD], radial diffusivity [RD]) were obtained using REKINDLE in ExploreDTI v4.8.6 (Leemans et al., 2009; Tax et al., 2015) whilst the estimates of intracellular volume fraction (ICVF) as a marker of neurite density were obtained using the NODDI MatLab Toolbox v0.9 (Zhang et al., 2012).

Multi-compartment relaxometry were calculated using the QUIT tools (Wood, 2018). The B1 field was estimated using DESPOT1-HIFI (Deoni, 2007) and then regularized by describing it as an 8th-order polynomial. The T1, T2 and off-resonance maps were then calculated (Deoni et al., 2003; Deoni, 2009), and the B1 and off-resonance maps used for the generation of the Myelin Water Fraction (MWF) maps using the echo-time corrected mcDESPOT equations (Bouhrara and Spencer, 2015).

#### 2.3.2 Generation of cortical and subcortical surface models

**Fig. 1:**
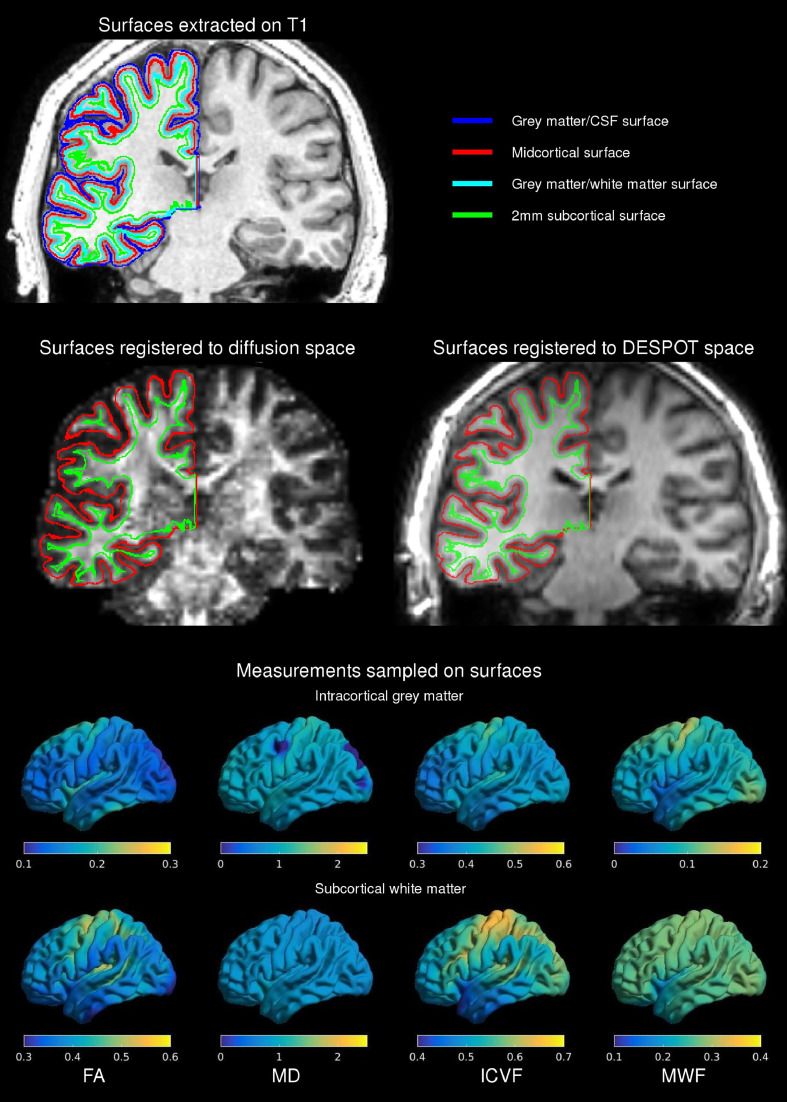
Image processing framework. Grey matter-white matter and grey matter-CSF cortical surfaces were extracted from the T1-weighted image (top, cyan and blue respectively) and midcortical and 2 mm subcortical surfaces were generated using a Laplacian potential (top, red and green respectively). These surfaces were registered to diffusion and DESPOT space using the FA image (middle left) and IR-SPGR (middle right) respectively. Measurements from diffusion and DESPOT scans were sampled along these surfaces (bottom). Examples of mean values in patients on the two surfaces are shown for diffusion (FA, MD, ICVF) and DESPOT (MWF) scans.

Surface models of the inner (grey matter-white matter) and outer (grey matter-CSF) cortical surfaces with 40,962 vertices per hemisphere were extracted (Fig. 1) (Kim et al., 2005). The CLASP algorithm iteratively warps a surface mesh to fit the grey-white matter boundary which is then expanded along a Laplacian map to model the grey matter-CSF surface. Surface extraction accuracy was visually verified and inaccuracies corrected. Surface-based registration based on cortical folding was used to improve intersubject correspondence in measurement location (Robbins et al., 2004).

Cortical grey matter was assessed along a surface placed at the midpoint between the inner and outer cortical surfaces (midcortical) and superficial white matter was examined along a surface running 2 mm below the grey-white matter boundary (2 mm subcortical). Both surfaces were generated using a Laplacian potential between the inner and outer surfaces and the white-grey matter interface and ventricular walls respectively as previously described (M Liu et al., 2016).

#### 2.3.3 Feature sampling

Linear transformations between the diffusion and DESPOT imaging spaces and the T1-weighted images in MNI space were calculated using the FA and IR-SPGR images respectively. The inverse of these transformations was used to map all surfaces generated on the T1-weighted images into the relevant native space to minimize data interpolation (Fig. 1).

FA, MD, AD, RD and ICVF (in diffusion space) and myelin water fraction (MWF) (in DESPOT space) were each sampled along the midcortical and 2 mm subcortical surfaces (Fig. 1).

Cortical thickness was calculated as the Euclidean distance between corresponding points on grey matter–white matter and grey matter–CSF surfaces (Kim et al., 2005).

### 2.4 Statistical analysis

Surface-based analyses were performed using the SurfStat Matlab toolbox (Worsley KJT et al., 2009). Prior to analysis, all measurements were surface-registered and smoothed using a diffusion kernel with a full-width-half-maximum of 20 mm. Patients’ hemispheric data were flipped such that the left side was ipsilateral to the focus. To minimize confounds from inter-hemispheric asymmetry, prior to flipping, measures at each vertex were normalized using a z-transformation with respect to the corresponding distribution in healthy controls (M Liu et al., 2016). The groups were matched for age and sex, and their additional inclusion as covariates did not affect the results of subsequent analyses. Findings are reported with family-wise error (FWE) correction of 0.05 using random field theory for non-isotropic images and a cluster defining threshold of 0.01 (Worsley et al., 1999).

The main effects for each parameter were established with vertex-wise Student's t-tests to map differences between patients and controls. Subsequently, the relationship between conventionally reported metrics (cortical thickness, anisotropy and diffusivity) and multi-compartment metrics (neurite density, MWF) was determined by assessing degree of overlap (Dice score) and through linear regression models.

Vertex-wise CSF partial volume estimates from a mixed tissue class model (Kim et al., 2005) were sampled in order to correct for partial volume effects (PVE). A linear model was fitted at each vertex v of the form P(v) ~ b0 + b1*PVE(v), where P(v) is the vertex-wise value of the measurement and PVE(v) is the CSF partial volume at the same vertex. The CSF-corrected measurement at the vertex P_c_(v) was calculated as the residual P_c_(v) = *P*(v) – (b0+b1*PVE(v)) as previously described (Bernhardt et al., 2018) and the group comparison was repeated. The same approach was used to correct conventional metrics for neurite density and MWF.

Further linear models explored the relationship between the multi-compartment parameters neurite density and MWF and the clinical variables hippocampal volume (Winston et al., 2013) and duration of epilepsy.

Further post-hoc analysis of the main findings was conducted with 10 mm FWHM smoothing to confirm robustness of the results. Scatterplots of the main positive relationships were generated from the means of the two parameters for each patient within the main cluster of differences in FA or MD identified between controls and patients (within the ipsilateral temporal lobe).

## 3 Results

### 3.1 Intracortical grey matter

**Fig. 2:**
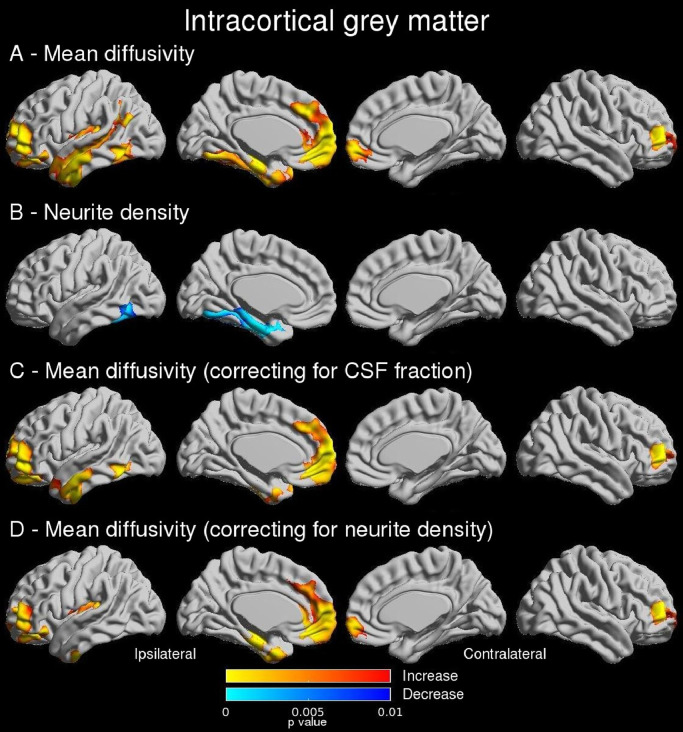
**I**ntracortical grey matter (main and regression findings). Group comparisons show that in patients mean diffusivity was increased in ipsilateral temporal and frontopolar regions (A) whilst reduced neurite density was more confined to mesial and basal temporal regions (B). Linear regression showed that increased mean diffusivity was related to both CSF fraction (C) and neurite density (D). Uncorrected p-values shown for significant clusters (defined by FWE 0.05, cluster threshold 0.01).

Compared to controls, patients demonstrated reduced neurite density confined to the ipsilateral mesial and basal temporal regions, including parahippocampal and fusiform gyri (Fig. 2B). Regions of increased MD were more extensive affecting the ipsilateral temporal pole, mesial and lateral temporal and frontopolar cortices (Fig. 2A), as well as contralateral orbitofrontal regions. AD and RD revealed identical findings (Supplementary Figure 1A-B). No significant group differences were demonstrated in FA or MWF.

As increased diffusivity could be driven by reduced neurite density (thus increased extracellular fluid) or tissue atrophy (thus increased CSF fraction), we explored these relationships with linear regression models. Diffusivity changes were associated with reduced neurite density in the ipsilateral temporal pole and lateral neocortex; to altered neurite density and CSF fraction in the fusiform gyrus; and primarily to CSF fraction in the parahippocampal gyrus (Figs. 2C and 2D).

Findings were similar with 10 mm FWHM smoothing (Supplementary Figure 3). Scatterplots showing the relationship between increased MD and neurite density, but a lack of relationship to MWF are shown in Supplementary Figure 4.

### 3.2 Subcortical white matter

**Fig. 3:**
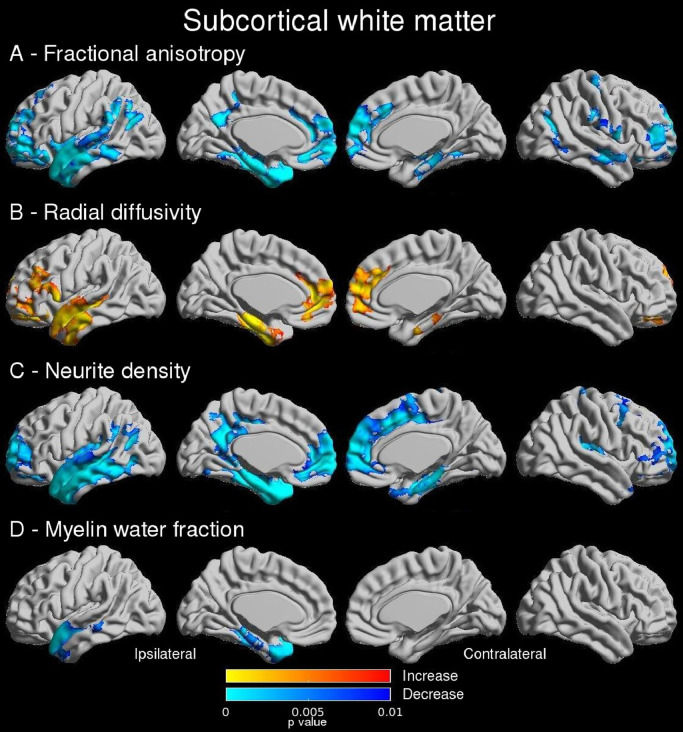
Subcortical white matter (main findings). Group comparisons show that in patients, bilateral reductions in FA were observed in temporal and frontopolar regions (A) with a similar distribution of increased RD (B) and reduced neurite density (C). Reduced myelin fraction (D) was more confined to the ipsilateral temporal lobe. Uncorrected p-values shown for significant clusters (defined by FWE 0.05, cluster threshold 0.01).

Bilateral reductions in FA encompassing the temporal pole, mesial and lateral temporal and prefrontal cortices were observed with stronger effects in the ipsilateral hemisphere (Fig. 3A). The primary change in diffusivity was an increase in RD with a similar but more limited distribution to FA (Fig. 3B) with 41.3% of vertices with reduced FA showing an increased RD. Differences in MD were minimal (Supplementary Figure 1C) whilst no difference in AD was observed. Reduction in neurite density demonstrated a similar but more widespread distribution of changes to those with FA and RD (Fig. 3C). A reduction in MWF was seen in the ipsilateral temporal pole and anterolateral temporal cortex (Fig. 3D).

**Fig. 4:**
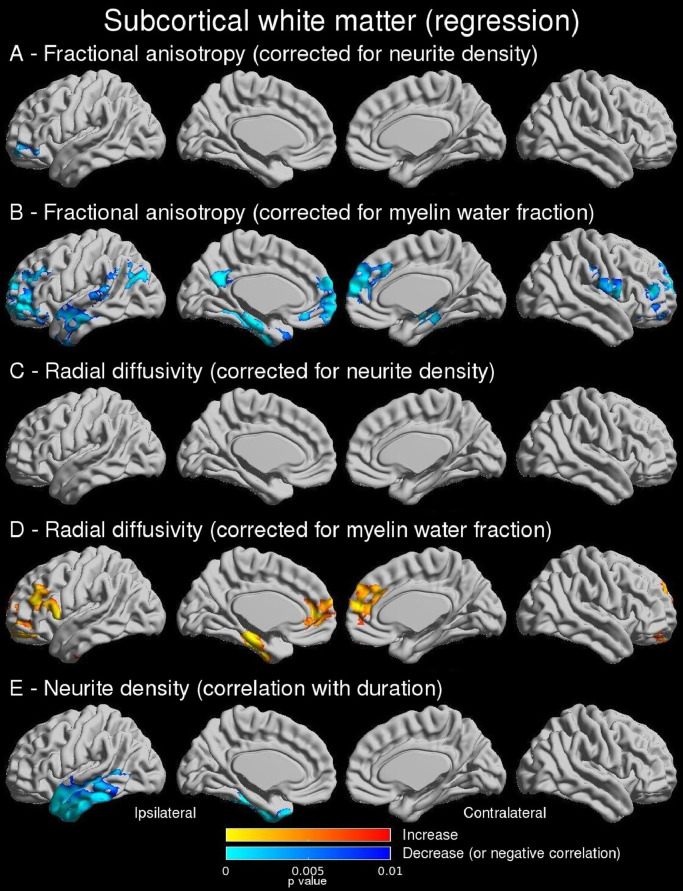
Subcortical white matter (regression findings). Linear regression showed that reduced FA in the ipsilateral temporal lobe is associated with axonal loss (A) with an additional relationship to altered myelination in the temporal pole and anterolateral temporal neocortex (B). A similar pattern was observed for the increase in RD (C,D). Neurite density in the temporal pole was more reduced with longer disease duration (E, shown with an outlier removed). Uncorrected p-values shown for significant clusters (defined by FWE 0.05, cluster threshold 0.01).

Using linear regression models, the reduction in FA was primarily associated with reduced neurite density (compatible with axonal loss) (Fig. 4A) whilst there was an additional role of myelination in the temporal pole and anterolateral temporal neocortex (Fig. 4B). The findings for RD were the same (Figs. 4C and 4D). Overall, 67.5% of vertices with reduced FA and 79.1% of vertices with increased RD also demonstrated reduced neurite density (Dice scores 0.61 and 0.47 respectively).

Findings were similar with 10 mm FWHM smoothing (Supplementary Figures 5 and 6). Scatterplots showing the relationship between reduced FA and both neurite density and MWF are shown in Supplementary Figure 7.

### 3.3 Relation to morphology

Compared to controls, cortical thickness was bilaterally reduced within the temporal lobe and centroparietal cortices in TLE (Supplementary Fig 2A). These changes were unrelated to any of the significant group changes described in grey or subcortical white matter (all Dice scores < 0.2, Supplementary Figure 2B–2C).

### 3.4 Clinical correlations

There was a significant correlation between reduced neurite density and disease duration in the ipsilateral lateral and basal temporal lobe (Fig. 4E) but there was no significant correlation with hippocampal volume. Changes in MWF were not related to disease duration or hippocampal volume. Findings were similar with 10 mm FWHM smoothing (Supplementary Figure 5E).

## 4 Discussion

### 4.1 Key findings

Multi-compartment imaging models offer an opportunity to study specific measures of tissue microstructure that more directly assess known histopathological alterations. We report the first study to combine markers of neurite density and myelination with surface-based techniques to better understand the changes in conventional diffusion-weighted metrics in TLE.

Increases in diffusivity throughout the neocortical grey matter of the ipsilateral temporal lobe were associated with reduced neurite density, with only a small relationship to CSF partial volume effects in basal regions. We did not demonstrate any role for altered MWF and extratemporal changes in frontopolar cortex were unrelated to neurite density.

Changes in diffusion parameters within subcortical white matter (reduced FA, increased RD) were more widespread and bilateral and associated with reduced neurite (axonal) density. An additional relationship to altered MWF was observed in the ipsilateral temporal pole and anterolateral temporal cortex. Changes were greater with longer disease duration.

Bilateral cortical thinning in the mesial temporal lobe and centroparietal cortices was unrelated to either neurite density or MWF.

Findings are robust to different levels of smoothing with similar findings using a post-hoc anlaysis at 10 mm FWHM smoothing compared to the pre-specified analysis at 20 mm FWHM.

### 4.2 Histological changes in TLE

The underlying histological changes being explored through neuroimaging are well characterized and the classical finding in TLE is hippocampal sclerosis. The most common subtype (ILAE type I) involves neuronal cell loss and gliosis in CA1 and CA4 regions (Blumcke et al., 2013). Neuronal loss and gliosis in TLE extends into parahippocampal and fusiform gyri and lateral temporal neocortex (Cavanagh and Meyer, 1956; Falconer et al., 1964; Kuzniecky et al., 1987; Nishio et al., 2000) and post-mortem studies show similar changes in frontal and occipital cortices (Margerison and Corsellis, 1966).

Microdysgenesis of the temporal neocortex in patients with TLE includes abnormally thick bundles of myelinated fibres in upper cortical layers with reduced neuronal density, subpial gliosis, neuronal ectopia and clustering and increased white matter neurons (Hardiman et al., 1988; Thom et al., 2000; Kasper et al., 2003; Eriksson et al., 2004). A greater extent of such developmental abnormalities is related to poorer seizure outcome after surgery (Hardiman et al., 1988; Thom et al., 2001).

Prior magnetic resonance studies identified temporopolar grey/white matter abnormalities including blurring in 32–68% of patients with TLE associated with HS (Choi et al., 1999; Meiners et al., 1999; Mitchell et al., 1999; Adachi et al., 2006) and that blurring is associated with loss of myelin on histological staining (Meiners et al., 1999; Garbelli et al., 2012). Thus alterations in both neurite density and myelination are observed.

### 4.3 Contribution of multi-compartment models

Conventional diffusion-weighted metrics assume a single tissue type within each voxel whereas multi-compartment models allow more specific study of the altered neurite density and myelination identified by histological studies. The NODDI model estimates neurite density (i.e. dendrites and axons) by considering each voxel as a combination of intracellular, extracellular and CSF fractions (Zhang et al., 2012) whilst the mcDESPOT model employed has three compartments – intra/extracellular, myelin and CSF – with myelin water fraction (MWF) yielding a measure of myelination (MacKay et al., 1994).

Reduced neurite density has been demonstrated in focal cortical dysplasia (Winston et al., 2014), but this technique has not been explored more systematically in TLE or in conjunction with surface-based analyses. In this paper, altered diffusivity in the ipsilateral temporal lobe grey matter and subcortical white matter was associated with widespread reduced neurite density as observed in histological studies. That subcortical white matter changes are greater with increasing duration concurs with the view that epilepsy is a neurodegenerative disorder with ongoing seizures leading to progressive neuronal loss (Bernasconi, 2016).

Previous imaging studies in patients with chronic refractory TLE have concentrated on identifying progressive atrophy through grey matter volumes (Bonilha et al., 2006) or cortical thickness (Bernhardt et al., 2009). Neurite density may represent a more sensitive and specific biomarker of neuronal damage from loss from ongoing seizures that deserves further study. Whether it relates to the progressive cognitive decline in TLE needs to be determined.

Altered MWF was important in only a restricted region of subcortical white matter involving the ipsilateral temporal pole and anterolateral temporal cortex, the same regions identified in previous histological studies (Meiners et al., 1999; Garbelli et al., 2012). Myelination has previously been studied by using T1 relaxometry as a proxy for cortical microstructure since it is sensitive to intracortical myelination (Bernhardt et al., 2018). Whilst data including post-mortem histology (Stuber et al., 2014), biophysical modelling (Koenig et al., 1990) and the correspondence between neocortical T1 and myeloarchitectural maps (Lutti et al., 2014; Waehnert et al., 2016) suggest that T1 values are related to grey matter myelin content, it is not a specific marker and may be affected by other factors.

Previous studies have demonstrated areas of cortical thinning (Bernhardt et al., 2010; Lin et al., 2007; McDonald et al., 2008; Whelan et al., 2018; Galovic et al., 2019) that dissociate from subcortical white matter changes (M Liu et al., 2016). This suggests independent pathological processes which have been postulated to result from the effects of seizure spread through thalamocortical pathways (Bernhardt et al., 2012). This paper provides further evidence that cortical thinning is unrelated to alterations in neurite density and MWF in cortical grey matter and subcortical white matter. Cortical thinning may therefore be related to factors such as gliosis that are not specifically addressed with the imaging parameters in this study.

### 4.4 Limitations and future work

This is the first study to combine multi-modal multi-compartment models with surface-based analyses to disentangle the contribution of neurite density and myelin water fraction to more conventional imaging parameters in TLE. The cohort size is accordingly modest and these findings should be replicated in a larger independent cohort. This would allow patients with left and right TLE to be separately analysed as whilst some papers report similar effects independent of laterality (Dabbs et al., 2012; M Liu et al., 2016), others report more diffuse changes in left (de Campos et al., 2016; Keller et al., 2002; Bonilha et al., 2007; Coan et al., 2009; Santana et al., 2010; Kemmotsu et al., 2011; Keller et al., 2012) or right TLE (Pail et al., 2010).

Although multi-compartment models are designed to provide biologically meaningful parameters derived from more plausible tissue models, limited data are available correlating imaging parameters with histology. The findings in this study agree very closely with previous histological data but the small number of patients who have undergone surgery preclude a detailed correlation of imaging and histological data. Future studies should address this.

Although the alterations of diffusion parameters and neurite density in subcortical white matter correlated with disease duration, this is a purely cross-sectional study and longitudinal studies are required to confirm whether this is related to progressive neuronal loss. It would also be informative to look at the correlation of the extent of these changes with neuropsychological data, such as working memory disruption, and seizure outcome.

## 5 Conclusions

We have shown that combining surface-based methods with multi-compartment imaging techniques can disentangle the contribution of neurite density and myelin water fraction to more conventional imaging parameters in patients with TLE. Diffusivity changes in ipsilateral temporal lobe grey matter and subcortical white matter primarily relate to reduced neurite density with an additional relationship to altered myelin water fraction in subcortical white matter.

Whilst these findings are in agreement with previous histological studies, histological confirmation is required to better understand parameters derived from multi-compartment models. The results open up the possibility of future studies to gain greater biological understanding of the pathophysiological changes underlying neuropsychological impairments and post-operative seizure outcome in TLE by combining these data and neuroimaging.

## CRediT authorship contribution statement

**Gavin P Winston:** Conceptualization, Methodology, Software, Formal analysis, Investigation, Resources, Data curation, Writing - original draft, Visualization, Funding acquisition. **Sjoerd B Vos:** Methodology, Software, Formal analysis, Investigation, Writing - review & editing. **Benoit Caldairou:** Methodology, Software, Formal analysis. **Seok-Jun Hong:** Methodology, Software. **Monika Czech:** Investigation, Data curation. **Tobias C Wood:** Methodology, Software, Writing - review & editing. **Stephen J Wastling:** Methodology, Software, Writing - review & editing. **Gareth J Barker:** Methodology, Software, Writing - review & editing. **Boris C Bernhardt:** Conceptualization, Methodology, Formal analysis, Software, Writing - review & editing, Supervision, Funding acquisition. **Neda Bernasconi:** Conceptualization, Resources, Writing - review & editing, Supervision, Funding acquisition. **John S Duncan:** Resources, Writing - review & editing, Supervision. **Andrea Bernasconi:** Conceptualization, Resources, Writing - review & editing, Supervision, Funding acquisition.

## Declarations of Competing Interest

None.

## Appendix Supplementary materials

Image, image 1

Image, image 2

Image, image 3

Image, image 4

Image, image 5

Image, image 6

Image, image 7
