# Supplementary figures and images for: Microstructural imaging in temporal lobe epilepsy: Diffusion imaging changes relate to reduced neurite density
Source: Neuroimage Clin. 2020 Feb 28;26:102231. doi: 10.1016/j.nicl.2020.102231 (PMC7063236; doi:10.1016/j.nicl.2020.102231)

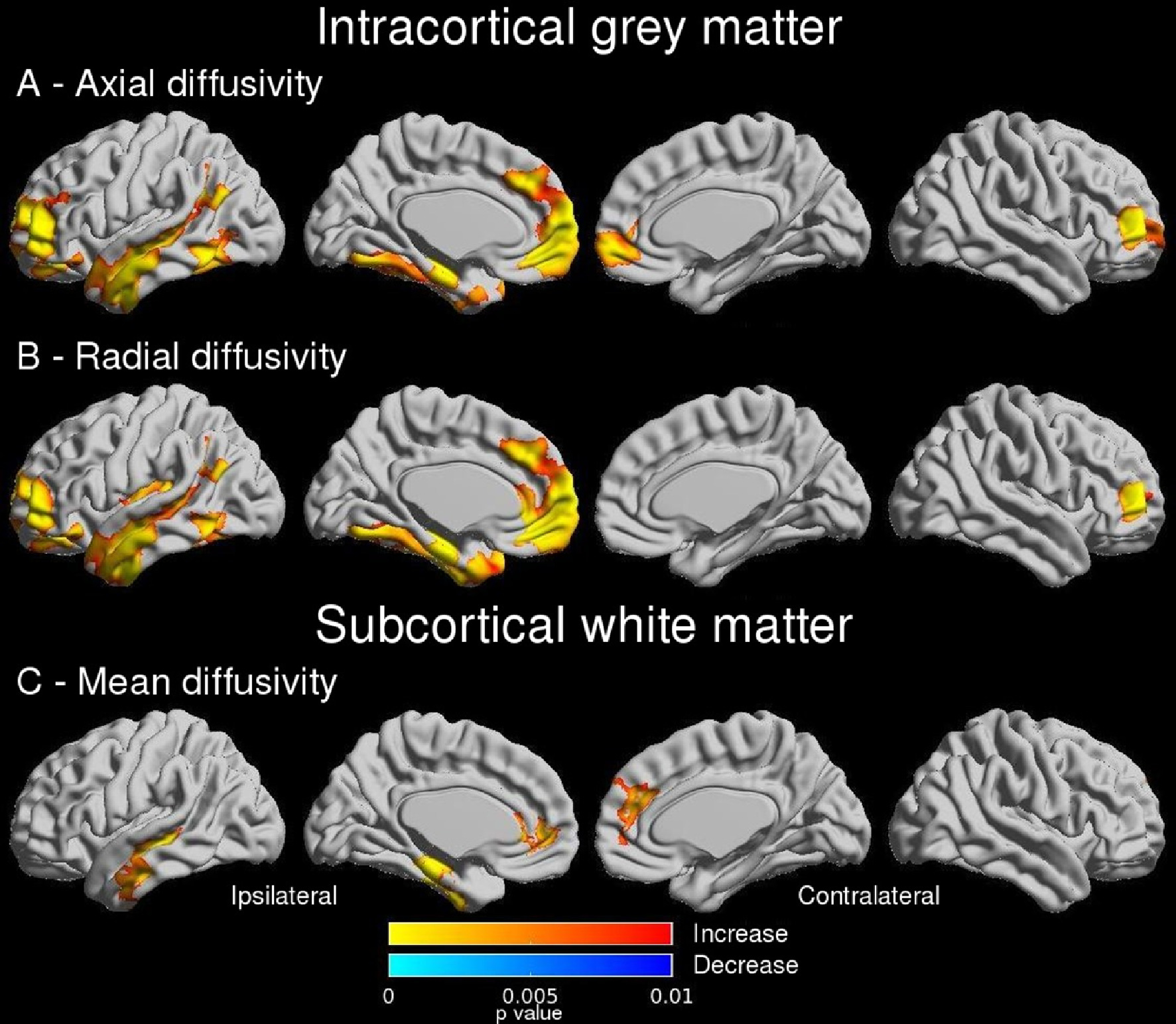

Supplement: Supplementary file 1 [file mmc1.jpg]

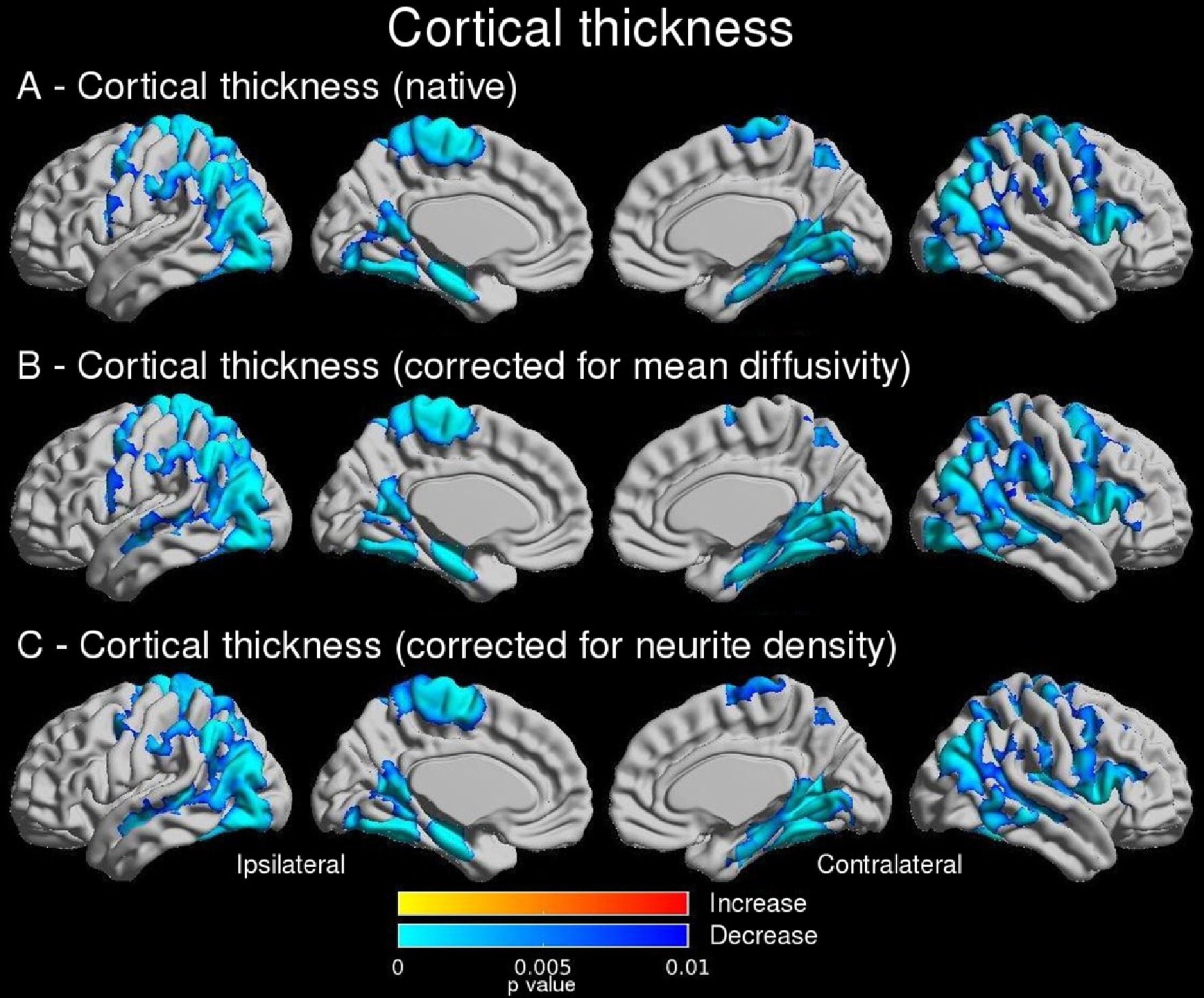

Supplement: Supplementary file 2 [file mmc2.jpg]

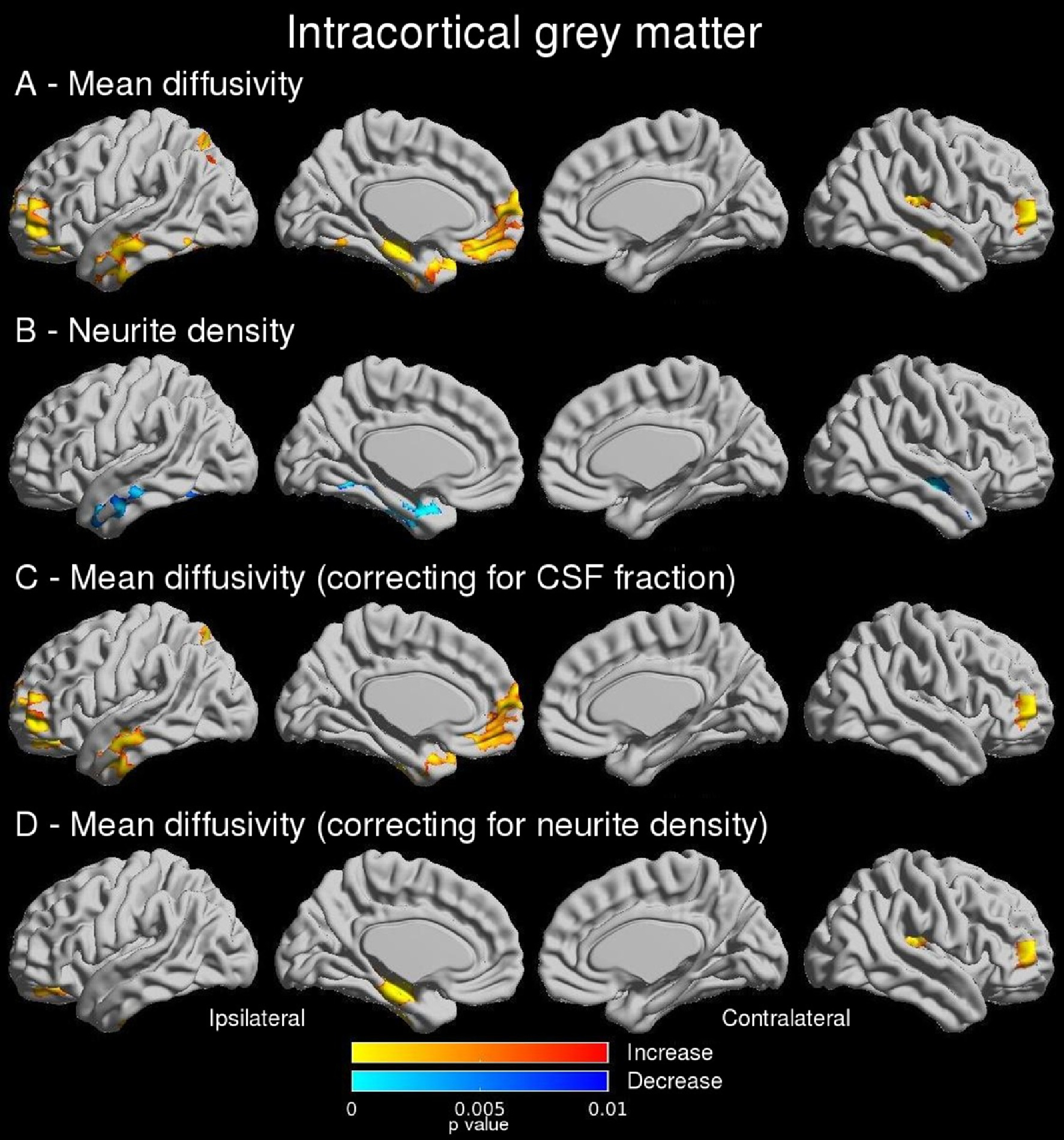

Supplement: Supplementary file 3 [file mmc3.jpg]

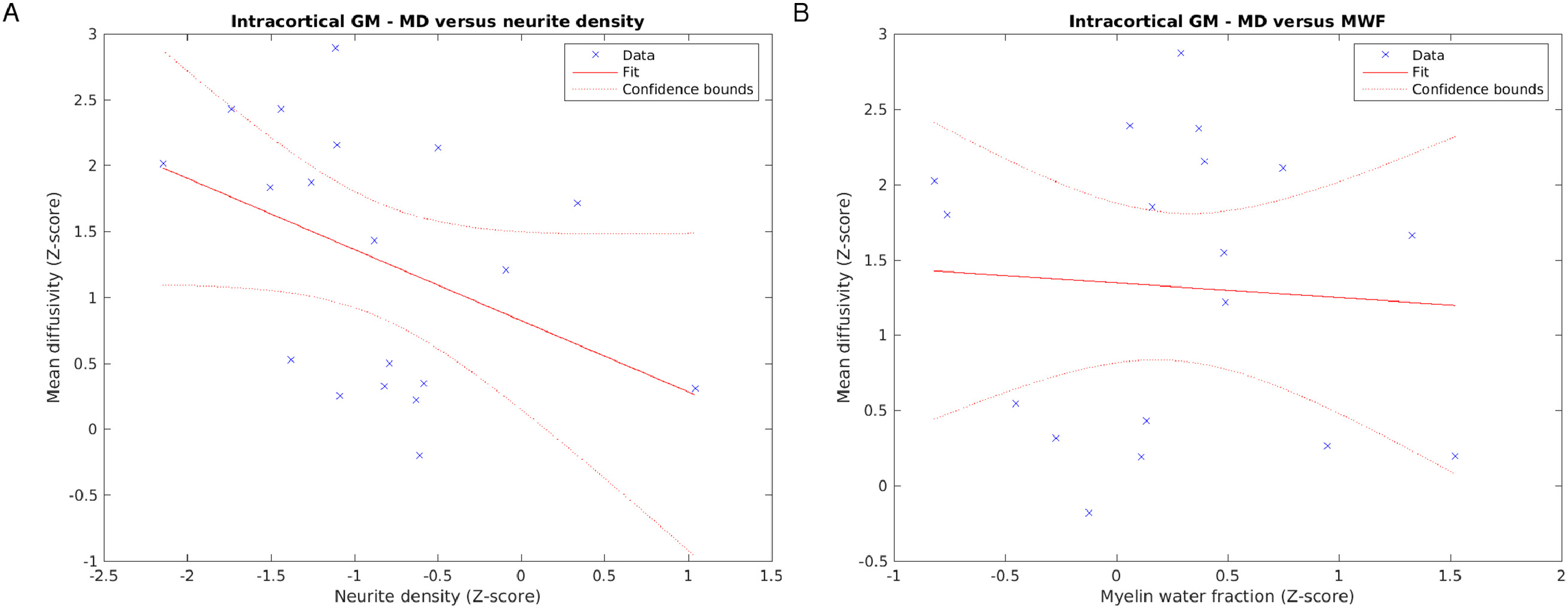

Supplement: Supplementary file 4 [file mmc4.jpg]

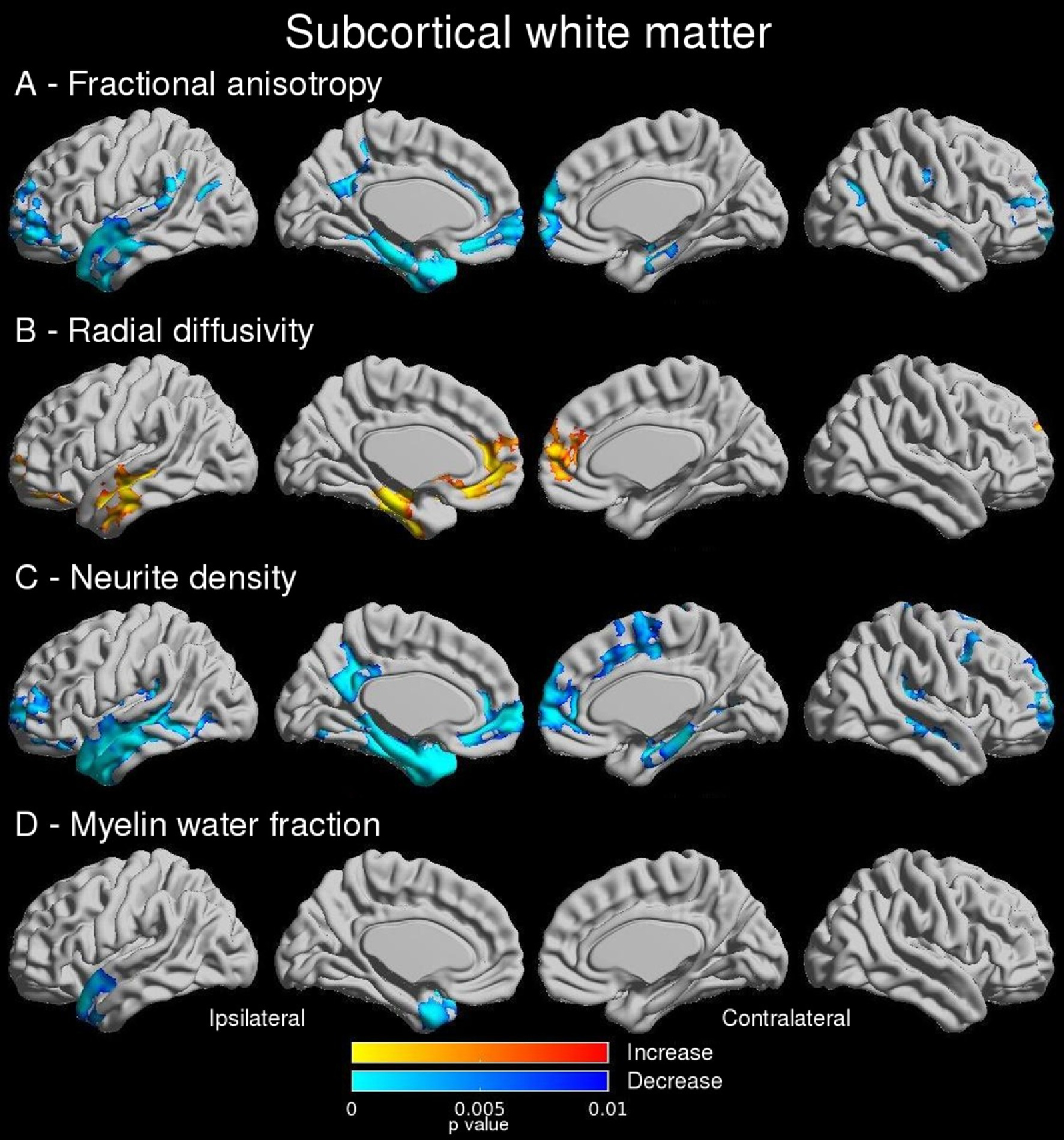

Supplement: Supplementary file 5 [file mmc5.jpg]

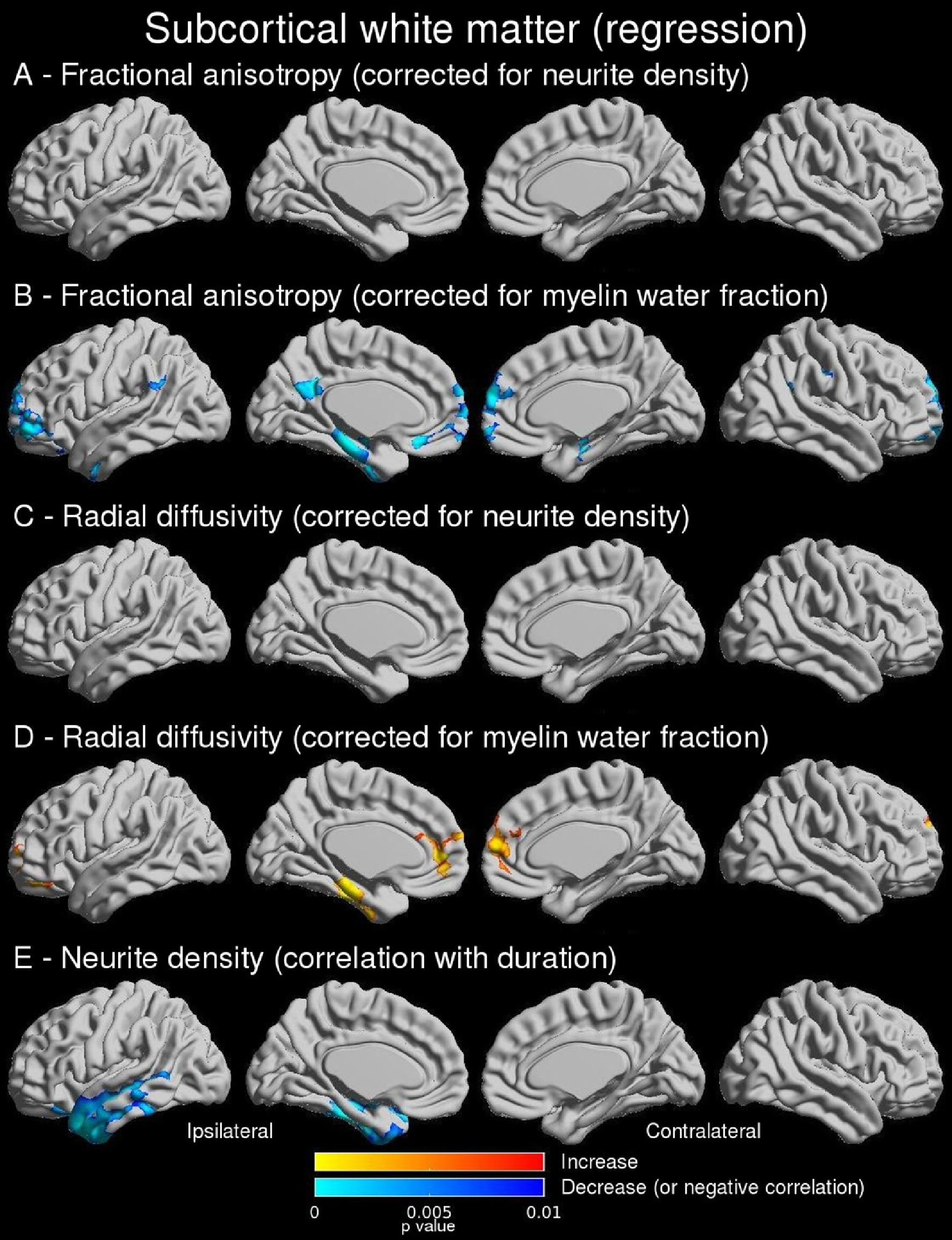

Supplement: Supplementary file 6 [file mmc6.jpg]

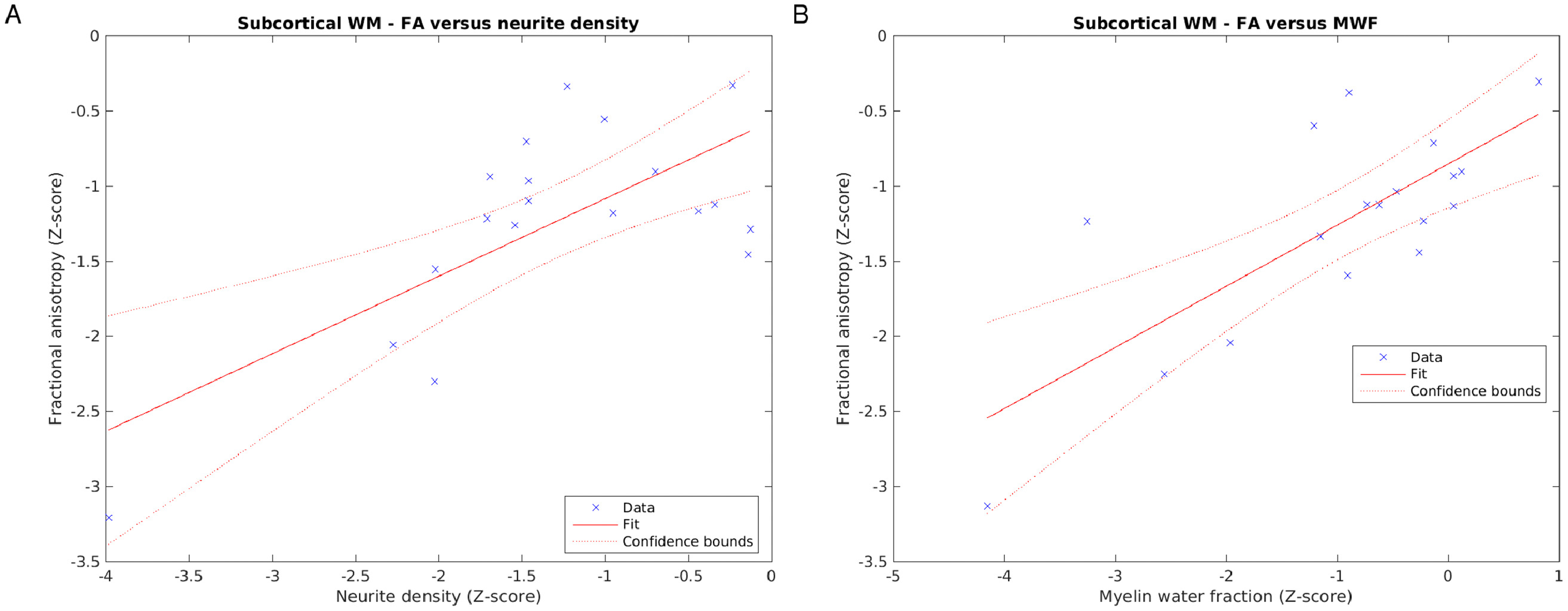

Supplement: Supplementary file 7 [file mmc7.jpg]
